# The p38 MAPK/MSK1/NF-κB axis in chronic prostatitis/chronic pelvic pain syndrome: a testable hypothesis for innate immune inflammatory amplification

**DOI:** 10.3389/fimmu.2026.1955957

**Published:** 2026-10-29

**Authors:** Yihang Shen, Baojun Ju

**Affiliations:** 1 Department of Andrology, The First Affiliated Hospital of Henan University of Chinese Medicine, Zhengzhou, China; 2 The First Clinical Medical College, Henan University of Chinese Medicine, Zhengzhou, China

**Keywords:** chronic prostatitis/chronic pelvic pain syndrome, inflammatory amplification, innate immunity, MSK1, neuroinflammation, NF-κB, oxidative stress, p38 MAPK

## Abstract

**Background:**

Chronic prostatitis/chronic pelvic pain syndrome (CP/CPPS) is a heterogeneous clinical syndrome that includes NIH category IIIA (inflammatory) and IIIB (noninflammatory) presentations, with pelvic pain, urinary symptoms, sexual dysfunction, and variable immune findings.

**Aim:**

To evaluate whether a p38 MAPK/MSK1/NF-κB network could amplify innate immune signaling in an immune-active subset of CP/CPPS.

**Evidence synthesis:**

Human evidence for coordinated p38, MSK1, and NF-κB activation in CP/CPPS is currently incomplete. p38 MAPK and NF-κB findings from prostatitis models and prostate-cell experiments provide disease-relevant support, whereas most MSK1, chromatin, mitochondrial-DAMP, and long-term inflammatory-memory links are extrapolated from non-prostatic inflammatory systems. MSK1 is activated by both p38 and ERK1/2 and has anti-inflammatory MSK1/2–DUSP1/IL-10 feedback functions; p65 Ser276 is one regulatory checkpoint among several NF-κB controls.

**Conclusions:**

The proposed MSK1-dependent amplification loop is a testable hypothesis rather than a demonstrated CP/CPPS mechanism. Patient-tissue phosphoproteomics, cell-resolved transcriptomics and ATAC-seq, and phenotype-stratified intervention studies are needed before therapeutic claims can be made. No proposed p38, MSK1, Nrf2, NOX, or NLRP3 strategy currently has established clinical efficacy in CP/CPPS.

## 1 Introduction

Chronic prostatitis/chronic pelvic pain syndrome (CP/CPPS) is a clinically heterogeneous syndrome characterized by pelvic pain, lower urinary tract symptoms, and sexual dysfunction, with a substantial impact on quality of life (1, 2). It includes NIH category IIIA (inflammatory) and IIIB (noninflammatory) presentations, and leukocyte counts do not reliably track symptom severity (3, 4). Phenotypic frameworks such as UPOINT and MAPP indicate overlapping urinary, psychosocial, organ-specific, infection-related, neurologic, and tenderness domains (5, 6). Treatment responses are variable and recurrence is common, suggesting that CP/CPPS cannot be explained by a single inflammatory stimulus or persistent infection (7). Increasing evidence implicates innate immune activation, inflammatory mediator production, oxidative stress, and neuroimmune dysfunction (8, 9). However, how initial triggers evolve into a persistent and self-sustaining chronic inflammatory state remains unresolved.

Recent advances indicate that chronic inflammation depends not only on signal initiation but also on amplification and stabilization. Damage-associated molecular patterns (DAMPs), oxidative stress-derived molecules, and pathogen-associated molecular patterns (PAMPs) activate pattern recognition receptors (PRRs), including Toll-like receptors (TLRs) and inflammasome-associated pathways, leading to mitogen-activated protein kinase (MAPK) and nuclear factor-κB (NF-κB) activation (10). Persistent signaling promotes tumor necrosis factor-α (TNF-α), interleukin-1β (IL-1β), and interleukin-6 (IL-6), while reactive oxygen species (ROS) accumulation can establish positive feedback loops that transform transient immune activation into a sustained inflammatory state (11–13). Therefore, identifying key regulatory nodes that connect innate immune sensing with inflammatory amplification is critical.

The p38 MAPK pathway plays a critical role in stress responses and inflammatory regulation; however, how it maintains persistent inflammatory transcriptional programs remains incompletely understood (14). Recent studies suggest that mitogen- and stress-activated protein kinase 1 (MSK1), a downstream effector of both p38 MAPK and ERK1/2, may contribute to inflammatory persistence by linking signal transduction with epigenetic regulation (15, 16). MSK1 can mediate histone H3 phosphorylation at Ser10 and Ser28 and chromatin remodeling, and can promote NF-κB p65 Ser276 phosphorylation, thereby enhancing inflammatory gene transcription (17, 18). However, MSK1 is not a p38-exclusive effector: MSK2 partially compensates for MSK1, the p38–MK2 branch can more directly control cytokine production in some settings, and MSK1/2 can also induce DUSP1 and IL-10 through CREB/ATF1, thereby limiting LPS-induced cytokine production (19, 20). In addition, p65 Ser276 is only one of several NF-κB regulatory checkpoints, alongside IKK-mediated IκBα degradation, p65 Ser536 phosphorylation, and noncanonical NF-κB signaling (21, 22). Thus, a network model is more accurate than a linear p38–MSK1–NF-κB axis, and the proposed axis should be regarded as a testable hypothesis rather than an established mechanism.

Based on this rationale, we propose that the p38 MAPK/MSK1/NF-κB axis is a candidate amplification module in an immune-active subset of CP/CPPS. We summarize evidence for its potential involvement in inflammatory mediator production, oxidative stress-associated feedback, and immune microenvironment remodeling, and discuss interactions with TLR4/MyD88 signaling, NLRP3 inflammasome activation, oxidative stress responses, and immune cell dysfunction. We also explore candidate therapeutic strategies targeting this network, while emphasizing that none has established clinical efficacy in CP/CPPS.

Throughout this review, evidence is interpreted at three levels: (i) demonstrated in human CP/CPPS samples; (ii) supported by prostatitis models or prostate-cell experiments; and (iii) extrapolated from non-prostatic inflammatory systems and therefore hypothesis-generating. Experimental autoimmune prostatitis (EAP), chronic nonbacterial prostatitis (CNP), and human CP/CPPS are related but non-equivalent evidence sources (23, 24). Accordingly, the proposed network should be tested in biomarker-defined, immune-active subgroups rather than assumed to explain CP/CPPS as a whole.

## 2 Remodeling of the inflammatory–oxidative stress microenvironment in chronic prostatitis

### 2.1 Formation of a persistent inflammatory microenvironment in chronic prostatitis

Although CP/CPPS lacks clear evidence of persistent infection, increasing studies indicate that aberrant immune activation and sustained inflammatory signaling are critical contributors to disease persistence and progression (9). During the pathological process of CP/CPPS, various stimuli, including infectious challenges, urinary reflux, mechanical injury, and tissue stress, may induce prostate epithelial cells and immune cells to release damage-associated molecular patterns (DAMPs), thereby activating innate immune responses mediated by pattern recognition receptors (PRRs) (25, 26). Among these receptors, Toll-like receptors (TLRs) are considered essential sensing systems that link tissue injury to inflammatory responses. Persistent or dysregulated activation of TLR signaling can further trigger downstream inflammatory pathways, including mitogen-activated protein kinase (MAPK) and nuclear factor-κB (NF-κB) signaling, thereby promoting the expression of pro-inflammatory mediators and maintaining the local inflammatory state (27).

Within the inflammatory microenvironment, immune cells, including macrophages, neutrophils, and T lymphocytes, play important regulatory roles. Studies using experimental autoimmune prostatitis (EAP) models have demonstrated significant infiltration of CD68-positive macrophages and CD4-positive T cells in prostate tissues, accompanied by increased expression of TNF-α, IL-1β, IL-6, and multiple chemokines (28, 29). In addition, abnormal activation of Th17 cells and increased IL-17A production have been implicated in immune-inflammatory regulation in CP/CPPS, further promoting inflammatory cell recruitment and tissue injury (30).

Importantly, chronic inflammatory states are not maintained by individual inflammatory mediators alone but rather arise from dynamic interactions among immune cells, inflammatory factors, and intracellular signaling pathways. Persistent activation of innate immune signaling promotes the release of inflammatory mediators, which in turn further enhances immune cell activation, establishing inflammatory positive feedback loops. These self-reinforcing networks gradually transform transient immune activation into a chronic inflammatory microenvironment with sustained inflammatory activity.

### 2.2 Oxidative stress as an amplifier of chronic inflammatory responses

Oxidative stress is not merely a passive consequence of inflammatory responses in CP/CPPS but rather represents an active signaling event involved in the regulation of inflammatory persistence. It interacts with immune activation to establish a mutually reinforcing amplification network. Under physiological conditions, the production of reactive oxygen species (ROS) is maintained in dynamic equilibrium with antioxidant defense systems. However, under persistent inflammatory stimulation, infiltrating macrophages, neutrophils, and damaged tissue cells generate excessive amounts of ROS. When ROS production exceeds the capacity of cellular antioxidant systems, oxidative stress is established (31). Excessive ROS can directly induce lipid peroxidation, protein oxidative modification, and DNA damage, thereby impairing prostate tissue homeostasis and function (32). However, within chronic inflammatory environments, the effects of ROS extend beyond oxidative injury. Increasing evidence indicates that ROS are not simply terminal products of inflammatory damage but also function as critical regulators of inflammatory signal amplification (33). ROS can enhance inflammatory transcriptional activity through activation of TLR4-associated signaling, p38 MAPK, and NF-κB pathways, while also promoting NLRP3 inflammasome activation, thereby establishing sustained inflammatory amplification responses (34).

Clinical studies have demonstrated that CP/CPPS patients exhibit elevated ROS levels in semen and prostate-associated samples, accompanied by impaired antioxidant capacity, including reduced activity of superoxide dismutase (SOD), catalase (CAT), and glutathione-related antioxidant systems (35). Furthermore, increased levels of oxidative damage markers, such as malondialdehyde (MDA), further support the involvement of oxidative stress in CP/CPPS progression.

Therefore, oxidative stress should not be considered merely an accompanying event of inflammatory injury in CP/CPPS. Instead, it may function as a critical amplification node connecting aberrant innate immune activation with persistent inflammation, providing upstream stimuli for the sustained activation of the p38 MAPK/MSK1/NF-κB inflammatory amplification module.

### 2.3 Inflammatory–oxidative stress coupling establishes a self-amplifying network in chronic prostatitis

Inflammation and oxidative stress are not independent pathological processes but instead interact through bidirectional regulation to establish a self-amplifying network, representing an important mechanism underlying the chronicity of CP/CPPS. Within the chronic inflammatory microenvironment, activated macrophages, neutrophils, and damaged tissue cells continuously generate excessive reactive oxygen species (ROS) through mechanisms including NADPH oxidase (NOX)-dependent pathways (36). Beyond serving as mediators of oxidative damage, ROS also function as important signaling molecules involved in inflammatory regulation by activating innate immune-associated pathways and promoting inflammatory persistence (37). On the one hand, ROS derived from inflammatory cells can enhance the activity of inflammatory signaling pathways, including TLR4/MAPK/NF-κB signaling, and promote NLRP3 inflammasome activation, thereby further inducing the release of inflammatory mediators such as IL-1β and IL-18. On the other hand, sustained expression of pro-inflammatory cytokines, including TNF-α, IL-1β, and IL-6, can further enhance ROS production, forming a positive feedback loop characterized by “inflammatory mediators–ROS–inflammatory signaling” amplification (9). This inflammatory–oxidative stress coupling network may transform transient immune activation into a chronic inflammatory state with self-sustaining properties.

Therefore, the reciprocal amplification between inflammation and oxidative stress not only represents an important basis for tissue injury in CP/CPPS but may also provide critical upstream stimuli for the persistent activation of the p38 MAPK/MSK1/NF-κB signaling axis. Through this mechanism, the axis may function as a central regulatory module linking aberrant innate immune activation with inflammatory transcriptional amplification.

## 3 Composition and biological functions of the p38 MAPK/MSK1/NF-κB signaling axis

### 3.1 The p38 MAPK signaling pathway in inflammatory regulation: evidence and gaps

p38 mitogen-activated protein kinase (p38 MAPK) is a critical member of the MAPK family and serves as an essential signal transduction module that senses extracellular stress signals and initiates inflammatory responses (38). A variety of danger-associated stimuli, including inflammatory cytokines (TNF-α and IL-1β), pathogen-associated molecular patterns (such as lipopolysaccharide, LPS), oxidative stress, and tissue injury signals, can activate p38 MAPK through upstream kinases MKK3 and MKK6, which induce dual phosphorylation within the Thr-Gly-Tyr (TGY) motif and subsequently trigger p38 MAPK activation (39). TAK1 activates p38 MAPK indirectly through MKK3/6 and MKK4, rather than by direct phosphorylation of p38 (40). Activated p38 MAPK phosphorylates multiple downstream effectors, including mitogen- and stress-activated protein kinase 1 (MSK1), transcription factors, and chromatin regulatory proteins, thereby converting extracellular danger signals into sustained nuclear transcriptional responses (14). During inflammatory regulation, p38 MAPK promotes the expression of pro-inflammatory genes, including TNF-α, IL-1β, and IL-6, and participates in diverse pathological processes such as immune cell activation, apoptosis regulation, and tissue remodeling (41).

Importantly, the biological function of p38 MAPK extends beyond the initial activation of inflammatory signaling and may contribute to the persistence and amplification of inflammatory responses. Within chronic inflammatory environments, continuous exposure to ROS, DAMPs, and inflammatory mediators may repeatedly activate p38 MAPK, allowing it to function as an important regulatory node linking innate immune sensing with inflammatory transcriptional programs (42). However, the precise role of p38 MAPK in CP/CPPS remains incompletely understood. Current evidence is largely derived from autoimmune disorders, infectious inflammatory models, and tissue injury models, whereas the dynamic activation patterns, cell-type-specific functions, and stage-dependent effects of p38 MAPK within the chronic low-grade inflammatory environment of CP/CPPS remain poorly characterized. In addition, p38 MAPK comprises multiple isoforms, including p38α/MAPK14, p38β, p38γ, and p38δ, and the relative contribution of these isoforms to inflammatory amplification in prostate tissue has not been defined (14). Whether the inhibitors used in prior studies are isoform-selective also requires clarification.

Furthermore, although p38 MAPK inhibitors have been investigated in various inflammatory diseases, their clinical efficacy has been less than expected, suggesting that targeting a single signaling node may be insufficient to reverse complex chronic inflammatory networks (43). Therefore, elucidating the downstream effectors responsible for p38 MAPK-mediated inflammatory persistence is critical for understanding inflammatory amplification mechanisms in CP/CPPS. Among these effectors, MSK1 may represent an important molecular bridge linking p38 MAPK activation with inflammatory transcriptional reprogramming.

### 3.2 MSK1: a critical bridge linking signal transduction with inflammatory transcriptional reprogramming

Mitogen- and stress-activated protein kinase 1 (MSK1) is an important downstream effector of both p38 MAPK and extracellular signal-regulated kinase (ERK1/2) signaling pathways, and is encoded by the RPS6KA5 gene (15). MSK1 contains dual kinase domains and can be directly phosphorylated and activated by either p38 MAPK or ERK1/2; phosphorylated ERK1/2 or p38 phosphorylates MSK1 at Ser360 and Thr581, which activates the C-terminal kinase domain (15). While both kinases can activate MSK1, the p38 branch is emphasized in this review because p38 MAPK is more directly linked to stress and innate immune signaling in inflammatory contexts, whereas ERK1/2 is also activated by growth factors and mitogenic stimuli (44). Unlike conventional inflammatory signaling molecules that primarily function as signal transmitters, MSK1 may further convert inflammatory stimuli into sustained transcriptional responses (45). Activated MSK1 can phosphorylate histone H3 at serine 10 (H3S10ph), promote alterations in chromatin accessibility, and enhance transcriptional activity at inflammation-associated gene loci (46). In addition, MSK1 regulates the activity of transcription factors such as CREB and ATF1, thereby cooperatively promoting the expression of inflammatory genes (47). Therefore, MSK1 may represent an important molecular bridge linking p38 MAPK activation with inflammatory transcriptional reprogramming.

However, MSK1 is not a p38-exclusive effector, and its function is context-dependent. MSK2 can partially compensate for MSK1 in inflammatory transcription, although MSK1 and MSK2 are not fully redundant and may have distinct substrate preferences (15). The p38–MK2 branch can more directly control cytokine production than MSK1 in some settings; MK2, a direct downstream substrate of p38, regulates LPS-stimulated TNF-α and IL-6 production through modulating the stability and translation of these mRNAs (48). Importantly, MSK1/2 can also exert anti-inflammatory effects: in macrophages, MSK1/2 induce DUSP1 and IL-10 through CREB/ATF1, thereby limiting LPS-induced cytokine production, and MSK1/2 double-knockout mice are hypersensitive to endotoxic shock (19). This dual role means that systemic MSK1 inhibition could remove protective feedback and potentially worsen inflammation (19). In addition, many earlier MSK1 studies relied on non-selective inhibitors such as H89, which also inhibits PKA, S6K1, and ROCK-II with comparable potency (49). The selectivity of these compounds should be considered when interpreting their results; SB-747651A is a more selective MSK1 inhibitor with an IC50 of 11 nM and only four off-targets at 1 µM, although it still inhibits PRK2, RSK1, p70S6K, and ROCK-II (50).

Accumulating evidence indicates that MSK1 participates in inflammatory amplification processes across multiple inflammatory disease models (51). For example, in colorectal adenocarcinoma, activation of the p38-MSK1 axis has been linked to prognostic outcomes, suggesting that MSK1 activity can be measured in human disease contexts (52). In neuroinflammation, MSK1 contributes to inflammatory pain via epigenetic and transcriptional alterations in the superficial spinal dorsal horn (53). These findings suggest that MSK1 may exert a conserved inflammatory amplification function across different inflammatory microenvironments, although its net effect depends on cell type and stimulus.

However, direct evidence regarding the involvement of MSK1 in CP/CPPS remains limited (24). Current studies have mainly focused on other inflammatory disease models, and it remains unclear whether MSK1 is aberrantly activated in CP/CPPS, whether its phosphorylation status correlates with disease severity, and whether MSK1 exhibits cell type-specific functions within the prostate inflammatory microenvironment (54). Moreover, given the partial functional redundancy between MSK1 and MSK2 (55), and the anti-inflammatory feedback mediated by MSK1/2–DUSP1/IL-10 (19), selective targeting of MSK1 alone may be influenced by compensatory mechanisms and could disrupt protective pathways. Therefore, defining the specific role of MSK1 in inflammatory persistence during CP/CPPS progression is essential not only for refining the proposed p38 MAPK/MSK1/NF-κB inflammatory amplification model but also for providing new theoretical insights into precision intervention strategies for chronic inflammatory diseases.

### 3.3 Epigenetic mechanisms by which MSK1 enhances NF-κB transcriptional activity

Nuclear factor kappa-B (NF-κB) is a central transcription factor regulating the expression of inflammatory genes, with the canonical form consisting of the p50/p65 heterodimer (22). NF-κB controls the transcription of a broad range of inflammation-associated genes, including pro-inflammatory cytokines such as TNF-α, IL-1β, and IL-6, as well as inflammatory mediators including MCP-1, IL-8, ICAM-1, VCAM-1, iNOS, and COX-2. Through these regulatory functions, NF-κB plays a critical role in innate immune activation and the maintenance of chronic inflammatory responses (56). Recent studies have demonstrated that NF-κB activity is not solely determined by its nuclear translocation but is also regulated by post-translational modifications and chromatin states. As an important effector molecule linking p38 MAPK signaling with NF-κB transcriptional programs, MSK1 may enhance NF-κB-mediated inflammatory gene expression through multiple mechanisms. Following activation, MSK1 translocates into the nucleus and directly phosphorylates the Ser276 residue of the NF-κB p65 subunit (18). This modification does not affect NF-κB nuclear translocation but facilitates the interaction between p65 and transcriptional coactivators CBP/p300, thereby enhancing the activity of the NF-κB transcriptional complex and promoting κB site-dependent inflammatory gene transcription (57).

However, p65 Ser276 phosphorylation should be treated as one regulatory checkpoint rather than a surrogate for total NF-κB activity (58). Canonical NF-κB signaling also depends on IKK-mediated IκBα degradation, p65 Ser536 phosphorylation, coactivator recruitment, and stimulus-specific noncanonical signaling (59). In addition, p65 Ser276 can be phosphorylated by other kinases, including PKA, MSK2, and IKKα, and the relative contribution of these kinases is cell-type- and stimulus-dependent (60). Therefore, the presence of p-p65 Ser276 alone does not establish MSK1-dependent NF-κB activation, and the relative contribution of these branches must be measured directly in CP/CPPS tissues and in relevant cell types.

Beyond regulating NF-κB transcriptional activity, MSK1 also influences the chromatin accessibility of inflammatory genes through epigenetic mechanisms. MSK1-mediated phosphorylation of histone H3 at Ser10 and Ser28 promotes the establishment of transcriptionally active chromatin states and may facilitate the recruitment of chromatin remodeling complexes, such as SWI/SNF, thereby increasing the accessibility of inflammation-associated promoters and enhancing the binding of transcription factors, including NF-κB, to target gene regions (17). Therefore, MSK1 may function as a “transcriptional amplifier” during inflammatory responses by converting transient inflammatory signals into sustained inflammatory gene expression programs.

Accumulating evidence supports the involvement of MSK1 in inflammatory regulation across multiple experimental models. For example, in *Mycobacterium avium*–induced macrophage inflammatory models, MSK1 activation contributes to the regulation of NF-κB- and CREB-dependent COX-2 expression, whereas MSK1 deficiency markedly reduces inflammatory gene transcription (61). In addition, MSK1-mediated histone modifications have been shown to participate in the regulation of inflammation-associated gene expression in other inflammatory cell models (15).

However, the contribution of MSK1-mediated NF-κB transcriptional enhancement to CP/CPPS remains unclear due to the lack of direct evidence. Several critical questions require further investigation: whether MSK1-dependent p65 Ser276 phosphorylation and chromatin remodeling determine the persistence of inflammatory gene expression in CP/CPPS, or whether they merely function as amplification mechanisms within an already activated inflammatory network. Furthermore, the therapeutic potential of targeting MSK1 requires evaluation in the context of disease stage and inflammatory burden. If MSK1 primarily contributes to inflammatory maintenance rather than inflammatory initiation, combined targeting of upstream immune sensing pathways and MSK1-mediated transcriptional amplification may provide greater therapeutic benefits than MSK1 inhibition alone.

### 3.4 The p38 MAPK/MSK1/NF-κB axis and the stabilization of inflammatory transcriptional programs: a hypothesis

The central feature of chronic inflammatory diseases is not merely the activation of inflammatory signaling pathways but rather the failure of effective inflammatory resolution, resulting in the long-term maintenance of pro-inflammatory transcriptional programs (62). The p38 MAPK/MSK1/NF-κB signaling axis may contribute to this process, but its role in stabilizing inflammatory states in CP/CPPS remains to be directly tested (63). Rather than simply promoting the expression of individual inflammatory mediators, this axis may integrate upstream danger signals, transcriptional regulation, and cellular state alterations, thereby contributing to self-sustaining regulatory networks (64).

Under acute inflammatory conditions, activation of p38 MAPK and NF-κB is generally transient and dynamically regulated, allowing inflammatory signaling to gradually return to baseline following elimination of the initial stimulus (65, 66). However, in the presence of persistent inflammatory stimuli, including infectious challenges, tissue injury signals, and oxidative stress, this signaling axis may undergo repeated activation, resulting in sustained inflammatory transcriptional activity. Particularly through the nuclear regulatory functions of MSK1, inflammation-associated genes may acquire increased transcriptional responsiveness, causing cells to exhibit enhanced responses to subsequent inflammatory stimuli (67). This phenomenon is consistent with the emerging concept of “inflammatory memory.” Inflammatory memory does not refer to the persistent activation of a single molecular pathway, but rather describes a cellular state in which previous inflammatory exposure induces epigenetic modifications, transcriptional network remodeling, and functional alterations, enabling cells to mount stronger or more prolonged inflammatory responses upon subsequent stimulation (68). However, direct evidence demonstrating whether MSK1-mediated transcriptional remodeling contributes to inflammatory memory formation in CP/CPPS remains lacking, representing an important question for future investigation.

Importantly, histone H3 Ser10 phosphorylation and p65 Ser276 phosphorylation are transient, signal-coupled events and do not by themselves constitute durable inflammatory memory (17, 21). Repeated TLR4 stimulation can also produce gene-specific endotoxin tolerance and transient silencing of pro-inflammatory genes, as shown by stimulus-dependent chromatin remodeling (69). This is the opposite of the amplification proposed here. A CP/CPPS memory claim would require persistent chromatin accessibility after stimulus withdrawal, a re-challenge design, longitudinal cell-specific ATAC-seq or ChIP-seq, and genetic or pharmacological manipulation of MSK1/2. Without such evidence, the concept of MSK1-mediated inflammatory memory in CP/CPPS remains a hypothesis, not an established mechanism.

Therefore, the p38 MAPK/MSK1/NF-κB axis may represent a candidate transition module linking “inflammatory stimuli” with “chronic inflammatory states”. Its persistent dysregulation may contribute to the transformation from transient immune activation to a stable chronic inflammatory microenvironment in CP/CPPS, but this possibility requires direct experimental validation. Reversal of such a proposed memory state is therefore a hypothesis, not an established therapeutic mechanism.

### 3.5 The p38 MAPK/MSK1/NF-κB axis mediates inflammatory–oxidative stress positive feedback amplification

The reciprocal interaction between inflammatory responses and oxidative stress is considered an important basis for the persistence of chronic inflammation (70). The p38 MAPK/MSK1/NF-κB signaling axis may serve as a candidate regulatory node linking these two pathological processes, although direct evidence in CP/CPPS is limited (71). On the one hand, this signaling axis can promote the activation of pro-inflammatory transcriptional programs and enhance the expression of inflammatory mediators, including TNF-α, IL-1β, and IL-6 (41, 72). These inflammatory cytokines can further stimulate immune cell activation and enhance NADPH oxidase (NOX) system activity, resulting in sustained reactive oxygen species (ROS) production and aggravation of oxidative stress (73). ROS are not merely terminal products of inflammatory injury but also function as signaling molecules capable of reactivating p38 MAPK and NF-κB pathways, thereby promoting the activation of inflammatory transcription factors and further amplifying inflammatory responses (74, 75). Thus, a reciprocal positive feedback loop has been proposed between “inflammatory mediator-induced ROS generation” and “ROS-mediated activation of inflammatory signaling pathways,” which may contribute to the long-term maintenance of inflammatory states in an immune-active subset of CP/CPPS.

However, most evidence for this feedback loop comes from non-prostatic inflammatory systems, and direct validation in CP/CPPS is lacking. In addition, MSK1/2 can also exert anti-inflammatory effects through DUSP1 and IL-10, and the p38–MK2 branch and ERK1/2–MSK1 pathway add further complexity; therefore, the net effect of this network depends on cell type and stimulus (19, 76, 77). Based on these findings, we propose a hypothetical inflammatory amplification model in which the p38 MAPK/MSK1/NF-κB axis may represent a candidate regulatory node connecting innate immune activation, oxidative stress imbalance, and persistent inflammatory remodeling. Within this network, MSK1 may increase the transcriptional responsiveness of inflammation-associated genes and potentially oxidative stress-related response genes, thereby reinforcing the stability of the inflammatory–oxidative stress network (15, 78). However, whether MSK1 directly regulates NOX family members, antioxidant defense systems, or ROS-responsive genes through epigenetic mechanisms remains to be experimentally validated.

Therefore, the p38 MAPK/MSK1/NF-κB axis may not merely function as a signal transduction pathway but may also represent a candidate regulatory mechanism maintaining inflammatory–oxidative stress feedback loops in CP/CPPS. This conceptual model provides a framework for understanding inflammatory persistence in CP/CPPS and offers potential directions for precision therapeutic strategies targeting inflammatory amplification networks, but it requires direct experimental validation before therapeutic claims can be made.

The proposed mechanism is illustrated in **Figure 1**. The molecular characteristics, regulatory functions of each component of the signal axis, as well as the current experimental basis for related studies, are summarized in **Table 1**.

**Figure 1:**
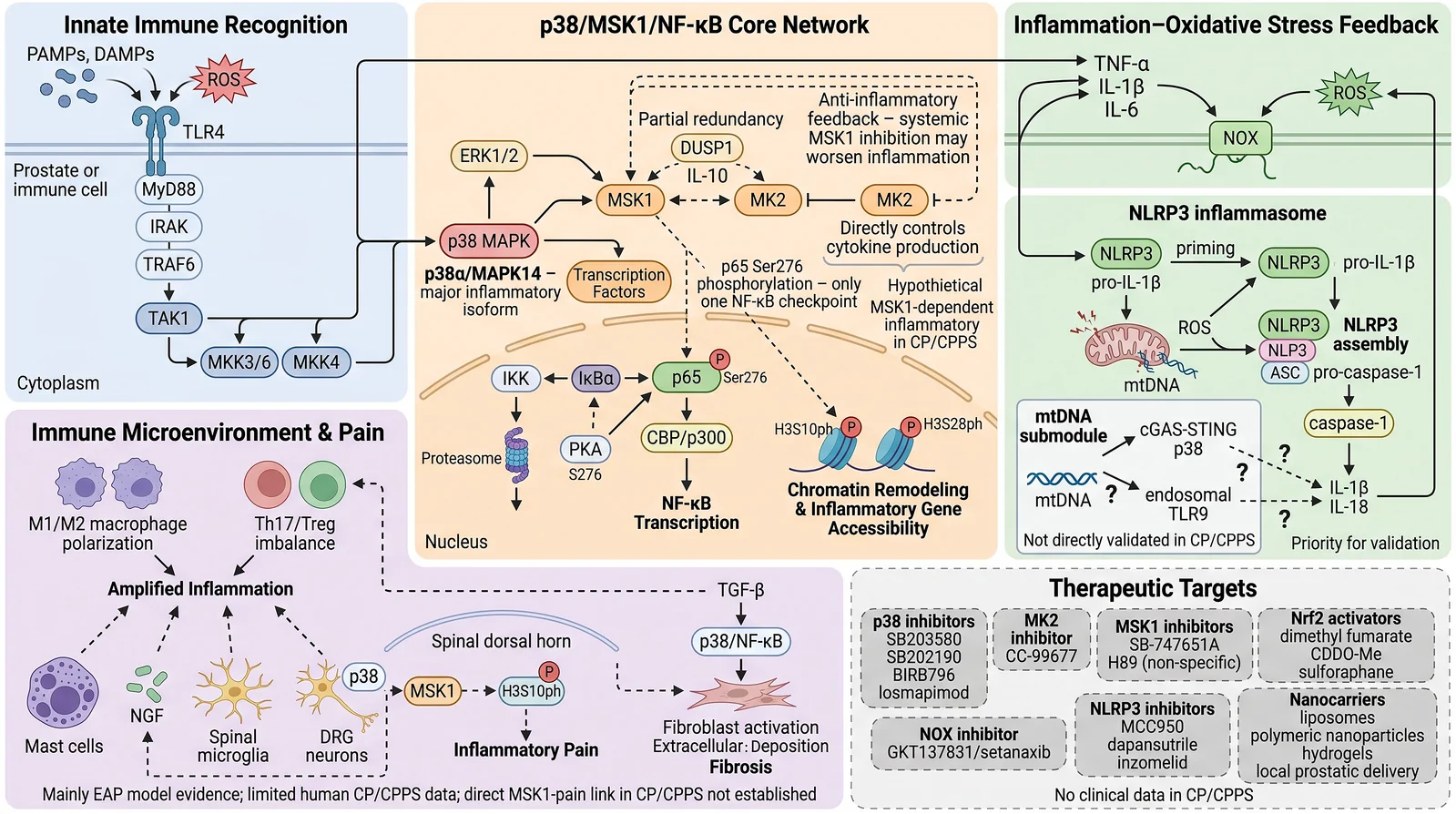
Proposed model of the p38 MAPK/MSK1/NF-κB axis as a candidate inflammatory amplification module in CP/CPPS. Persistent inflammatory stimuli activate innate immune sensing pathways, including TLR4/MyD88 and inflammasome-related signaling, leading to TAK1 activation; TAK1 activates p38 MAPK indirectly through MKK3/6 and MKK4. Activated p38 MAPK promotes MSK1 activation, which enhances NF-κB-dependent inflammatory transcription through p65 Ser276 phosphorylation and potential chromatin remodeling. MSK1 is also activated by ERK1/2, and MSK2 partially compensates for MSK1; the p38–MK2 branch can more directly control cytokine production. MSK1/2 can also induce DUSP1 and IL-10, limiting LPS-induced cytokine production. The resulting inflammatory mediators contribute to immune activation and oxidative stress accumulation. ROS and mtDNA further activate p38 MAPK/NF-κB signaling, forming a proposed positive feedback loop that may sustain chronic inflammatory states. NLRP3 inflammasome activation, macrophage polarization, Th17/Treg imbalance, mast cell activation, and neuroinflammation may also contribute to CP/CPPS chronicity. Solid arrows denote pathways supported by CP/CPPS or prostatitis models/prostate cells. Dashed arrows denote extrapolated or hypothetical links not yet demonstrated in human CP/CPPS tissue. Question marks indicate key unanswered questions. MSK1-dependent inflammatory memory remains hypothetical and would require re-challenge designs, ATAC-seq/ChIP-seq, and MSK1/2 manipulation; repeated TLR4 stimulation may instead induce endotoxin tolerance, which is opposite to amplification. Direct mtDNA-TLR9/STING signaling and its relationship to p38 MAPK/NF-κB activation in CP/CPPS have not been validated and represent a priority for future investigation. No p38, MSK1, Nrf2, NOX, or NLRP3-targeted strategy currently has established clinical efficacy in CP/CPPS.

**Table 1:** Evidence supporting the p38 MAPK/MSK1/NF-κB signaling axis in inflammatory amplification.

Signaling component	Molecular function	Evidence in inflammatory regulation	Evidence source	Direct or indirect	CP/CPPS validation status
p38 MAPK (p38α/MAPK14)	Stress-responsive kinase activated by inflammatory stimuli and oxidative stress; phosphorylates MSK1, transcription factors, and chromatin regulatory proteins	Activated by TNF-α, IL-1β, LPS, and ROS; regulates expression of TNF-α, IL-1β, and IL-6; increased p38 phosphorylation observed in chronic prostatitis models	Prostatitis models, prostate cells, non-prostatic inflammatory systems	Direct for p38 activation in prostatitis models; indirect for causal role in CP/CPPS chronicity	Activation observed in CNP/EAP models; causal role in human CP/CPPS not established
MSK1 (RPS6KA5)	Downstream kinase activated by p38 MAPK and ERK1/2; phosphorylates histone H3 at Ser10/Ser28 and p65 at Ser276	Mediates chromatin remodeling and NF-κB-dependent inflammatory transcription; participates in inflammatory amplification in gingival epithelial cells, airway smooth muscle cells, and macrophages	Non-prostatic inflammatory systems; prostate tissue largely uninvestigated	Indirect	Direct evidence in human CP/CPPS lacking
MSK2	Partially compensates for MSK1; may have distinct substrate preferences	Regulates inflammatory transcription; MSK1/MSK2 double knockout increases endotoxic shock sensitivity	Non-prostatic inflammatory systems	Indirect	Not examined in CP/CPPS
MK2	Direct downstream substrate of p38; regulates stability and translation of TNF-α and IL-6 mRNA	Controls LPS-stimulated TNF-α and IL-6 production more directly than MSK1 in some settings	Non-prostatic inflammatory systems	Indirect	Not examined in CP/CPPS
DUSP1/IL-10	Anti-inflammatory negative feedback induced by MSK1/2 through CREB/ATF1	Limits LPS-induced cytokine production; loss of this feedback worsens inflammation	Non-prostatic inflammatory systems	Indirect	Not examined in CP/CPPS
NF-κB p65	Central transcription factor controlling inflammatory gene expression (TNF-α, IL-1β, IL-6, MCP-1, IL-8, ICAM-1, VCAM-1, iNOS, COX-2)	Canonical NF-κB signaling depends on IKK-mediated IκBα degradation, p65 Ser536 phosphorylation, and coactivator recruitment	Prostatitis models, prostate cells, non-prostatic inflammatory systems	Direct for NF-κB activation; indirect for MSK1-dependent regulation	NF-κB activation observed in CP/CPPS models; MSK1-dependent branch unverified
p65 Ser276	Enhances NF-κB transcriptional activity via CBP/p300 recruitment	One regulatory checkpoint among several NF-κB controls; can also be phosphorylated by PKA, MSK2, and IKKα	Non-prostatic inflammatory systems	Indirect	Not measured in human CP/CPPS tissue
Histone H3 Ser10/Ser28	Epigenetic regulation of inflammatory gene accessibility	MSK1-dependent chromatin remodeling facilitates inflammatory gene transcription	Non-prostatic inflammatory systems	Indirect	Proposed mechanism; epigenetic validation required

## 4 Candidate mechanisms by which the p38 MAPK/MSK1/NF-κB axis may contribute to CP/CPPS chronicity

The core feature of chronic prostatitis/chronic pelvic pain syndrome (CP/CPPS) is not simply an excessive inflammatory response, but rather the failure of the local immune microenvironment to restore homeostasis, resulting in persistent activation of inflammatory signaling and progressive tissue dysfunction (79). However, how distinct pathological events interact to establish a sustained inflammatory amplification network remains incompletely understood. In this review, we propose that the p38 MAPK/MSK1/NF-κB signaling axis may serve as an important regulatory hub linking innate immune stimulation with chronic inflammatory maintenance. Rather than merely mediating transient inflammatory mediator release, this axis may integrate inflammatory signaling, oxidative stress responses, and immune cell functional states, thereby promoting the transition from acute inflammatory responses to a stable chronic inflammatory state in CP/CPPS.

### 4.1 Innate immune sensing and the p38 MAPK/MSK1/NF-κB axis: a proposed initiation of inflammatory amplification

The establishment of persistent inflammatory states in CP/CPPS is initially dependent on sustained activation of local innate immune responses (9). Prostate epithelial cells, macrophages, and other immune cells can recognize pathogen-associated molecular patterns (PAMPs) and damage-associated molecular patterns (DAMPs) through pattern recognition receptors (PRRs), thereby initiating inflammatory signaling cascades (10). Among these pathways, Toll-like receptor 4 (TLR4)/MyD88 signaling is considered an important link between external stimuli and inflammatory activation (27). In CP/CPPS, bacterial components or endogenous molecules released during tissue injury may activate TLR4, which subsequently recruits the adaptor protein MyD88 and initiates downstream signaling cascades involving the recruitment and phosphorylation of interleukin-1 receptor-associated kinases (IRAKs) and TNF receptor-associated factor 6 (TRAF6), ultimately leading to activation of transforming growth factor-β-activated kinase 1 (TAK1) (80). Activated TAK1 activates p38 MAPK indirectly through MKK3/6 and MKK4, rather than by direct phosphorylation of p38 (40). This MKK3/6- and MKK4-dependent step is required for p38 MAPK activation, but its specific involvement in CP/CPPS has not been directly tested (81).

TLR4 signaling not only activates p38 MAPK but also promotes NF-κB nuclear translocation, resulting in enhanced transcription of pro-inflammatory cytokines, including TNF-α, IL-1β, and IL-6 (63). These inflammatory mediators can subsequently function as autocrine or paracrine signals to further activate p38 MAPK signaling, thereby reinforcing inflammatory amplification (82). Animal studies have demonstrated that chronic nonbacterial prostatitis (CNP) models exhibit marked activation of p38 MAPK signaling in prostate tissues, accompanied by increased expression of pro-inflammatory mediators such as TNF-α and IL-1β, suggesting that p38 MAPK-associated inflammatory signaling may contribute to disease progression (24). However, these findings derive from animal models, and their correspondence to human CP/CPPS remains incompletely established. Experimental autoimmune prostatitis (EAP), chronic nonbacterial prostatitis (CNP), and human CP/CPPS are related but non-equivalent evidence sources, and model validity should be considered when interpreting these data.

Human data on cytokines in prostatic secretions and semen in CP/CPPS are available but heterogeneous (83). Elevated seminal levels of TNF-α, IL-1β, IL-6, and IL-8 have been reported in some CP/CPPS cohorts, particularly in NIH category IIIA patients, whereas category IIIB patients often show no clear inflammatory cytokine elevation (84). These findings support the presence of an immune-active subgroup within CP/CPPS but do not establish a causal role for the p38 MAPK/MSK1/NF-κB axis in disease chronicity. In addition, leukocyte counts in expressed prostatic secretions and semen do not reliably correlate with symptom severity, further indicating that inflammatory markers and clinical phenotype are dissociated in CP/CPPS.

Within this process, the p38 MAPK/MSK1/NF-κB axis may function as a candidate signal integration module rather than an established driver. Following activation of p38 MAPK by upstream immune sensing pathways, MSK1 activation may be subsequently enhanced, thereby potentiating NF-κB-mediated inflammatory transcriptional programs and transforming transient inflammatory responses into sustained activation states. However, direct evidence for coordinated p38, MSK1, and NF-κB activation in human CP/CPPS tissue is currently lacking. The precise upstream triggers responsible for persistent activation of the p38 MAPK/MSK1/NF-κB axis in CP/CPPS remain controversial (85). Since most patients lack evidence of persistent infection, factors driving long-term activation of this axis may extend beyond microbial stimulation and may include endogenous danger signals generated by urinary reflux, tissue injury, oxidative stress, and neuroimmune dysregulation (86). Therefore, elucidating the upstream triggers of this signaling axis represents an important direction for future precision therapeutic interventions, but current evidence remains hypothesis-generating.

### 4.2 The p38 MAPK/MSK1/NF-κB axis and the NLRP3 inflammasome: a proposed cooperative mechanism in CP/CPPS

A defining feature distinguishing chronic inflammation from acute inflammatory responses is the failure of effective mechanisms responsible for inflammatory resolution (87). In CP/CPPS, the p38 MAPK/MSK1/NF-κB axis may contribute to inflammatory persistence by promoting inflammasome activation and amplifying innate immune responses, but this remains a hypothesis.

The NLRP3 inflammasome represents a critical platform linking innate immune sensing with inflammatory amplification (88). Its activation generally requires two sequential steps. First, NF-κB signaling induces the expression of NLRP3 and inflammation-associated molecules, including pro-IL-1β, thereby providing the priming signal required for inflammasome activation (89). Subsequently, ROS accumulation, mitochondrial dysfunction, and cellular stress signals promote NLRP3 inflammasome assembly, resulting in caspase-1 activation and the maturation and release of IL-1β and IL-18 (90). These mechanisms are well established in other inflammatory systems. In CP/CPPS, NLRP3 inflammasome components have been detected in expressed prostatic secretions of patients and correlate with disease severity, although functional activation and causal contribution to disease chronicity require further validation (91).

Within this process, the p38 MAPK/MSK1/NF-κB axis may serve as a candidate regulatory link connecting inflammatory transcriptional activation with inflammasome activation. On the one hand, NF-κB enhances the expression of inflammasome-associated genes, thereby establishing the molecular basis for NLRP3 activation (92). On the other hand, cytokines released from activated inflammasomes, particularly IL-1β, may further activate MAPK/NF-κB signaling pathways, generating a proposed sustained inflammatory amplification loop (93). However, whether this loop operates in CP/CPPS has not been directly tested. Therefore, during CP/CPPS chronicity, the p38 MAPK/MSK1/NF-κB axis may not function independently but rather interact with the NLRP3 inflammasome to establish a coordinated inflammatory network. Through this interaction, transient inflammatory activation may gradually evolve into a persistent inflammatory state characterized by long-term maintenance of local immune activation, but this model requires experimental validation in CP/CPPS.

### 4.3 Oxidative stress-driven mitochondrial damage: a proposed amplification loop in CP/CPPS

Oxidative stress is an important feature of persistent inflammation in CP/CPPS, and ROS also function as signaling molecules involved in inflammatory network regulation (33). Under chronic inflammatory conditions, persistent activation of the p38 MAPK/MSK1/NF-κB axis may promote pro-inflammatory cytokine expression, enhance NADPH oxidase activity, and facilitate sustained ROS production (44, 94). However, whether this axis directly drives mitochondrial damage in CP/CPPS has not been demonstrated.

Excessive ROS accumulation can induce mitochondrial dysfunction, including membrane potential collapse, impaired ATP production, and mitochondrial DNA (mtDNA) damage (95). Under sustained oxidative stress, mitochondrial integrity becomes compromised, and mtDNA can be released into the cytoplasm or extracellular environment through mPTP opening, VDAC1 oligomerization, BAX/BAK macropores, or mitochondrial-derived vesicles (96, 97). These mechanisms are well established in other inflammatory systems, but direct evidence for mtDNA release in CP/CPPS is lacking (98, 99).

Released mtDNA can function as a damage-associated molecular pattern (DAMP) and activate innate immune sensing pathways (100). Depending on its localization, mtDNA may amplify inflammatory signaling through three major pathways: (1) the cGAS–STING pathway, in which cytosolic mtDNA activates STING and downstream TBK1–IRF3 and NF-κB signaling (101); (2) the NLRP3 inflammasome pathway, in which oxidized mtDNA promotes caspase-1 activation and IL-1β/IL-18 release (102); and (3) the TLR9 pathway, in which mtDNA activates NF-κB and MAPK signaling through MyD88 (103). These pathways are interconnected through shared downstream nodes, particularly NF-κB and p38 MAPK (104).

In the TLR9 pathway, mtDNA recognition activates the MyD88–IRAK–TRAF6–TAK1 cascade, leading to NF-κB and MAPK activation (105). In the cGAS–STING pathway, STING activation triggers MAPK signaling, and cGAS/STING can recruit MyD88-dependent mechanisms to induce p38-dependent inflammatory responses; however, this link is context-specific and has not been established in CP/CPPS (106). In the NLRP3 pathway, IL-1β can further stimulate both MAPK and NF-κB signaling, whereas IL-18 activates MAPK signaling and can activate NF-κB in a cell-type-dependent manner (107). Therefore, these pathways and the p38 MAPK/MSK1/NF-κB axis may form a bidirectional cross-talk network: mtDNA-induced TLR9, cGAS–STING, and NLRP3 pathways can enhance p38 MAPK and NF-κB activation, while inflammatory cytokines and oxidative stress driven by this axis may aggravate mitochondrial damage and promote additional mtDNA release; however, this bidirectional loop has not been directly tested in CP/CPPS.

This proposed positive feedback loop may contribute to the long-term maintenance of a pathological inflammatory cycle in CP/CPPS, but it remains a hypothesis. Future studies should prioritize direct measurement of mtDNA release, TLR9 and cGAS–STING activation, and their relationship to p38 MAPK/NF-κB signaling in human CP/CPPS tissue and relevant models.

### 4.4 Immune microenvironment remodeling: macrophage polarization and T-cell immune imbalance in CP/CPPS

Beyond regulating inflammatory mediator production, the p38 MAPK/MSK1/NF-κB axis may also contribute to the establishment of the inflammatory microenvironment in CP/CPPS by modulating immune cell functional states, although this remains a hypothesis. Macrophages are important regulators of local immune responses within the prostate (108). Inflammatory stimuli can induce macrophages to acquire a pro-inflammatory phenotype through TLR4/NF-κB signaling, leading to the release of inflammatory mediators, including TNF-α and IL-1β, thereby promoting tissue injury and inflammatory persistence (109). In experimental autoimmune prostatitis (EAP) models, peroxiredoxin 5 (Prdx5) has been shown to activate the TLR4/NF-κB pathway in a ROS-dependent manner, promoting inflammatory macrophage phenotypes and aggravating prostate epithelial cell injury (110). However, this evidence derives from EAP models, and its correspondence to human CP/CPPS remains incompletely established.

Human studies on macrophage polarization in CP/CPPS are limited. Some studies have reported increased M1-like macrophage markers and pro-inflammatory cytokines in expressed prostatic secretions and seminal plasma from CP/CPPS patients, in CP/CPPS patients, although stratified data by NIH category are limited (111). However, these findings are heterogeneous, and whether M1/M2 polarization is causally linked to symptom severity or disease chronicity has not been established (111). Therefore, the proposed role of the p38 MAPK/MSK1/NF-κB axis in macrophage polarization in CP/CPPS remains speculative, and the contribution of MSK1 specifically has not been examined.

Adaptive immune dysregulation represents another factor potentially involved in CP/CPPS chronicity (9). An imbalance between Th17 and regulatory T cells (Tregs) has been implicated in disease-associated immune abnormalities, in which IL-17-driven inflammatory responses may further enhance the local inflammatory environment (112). The p38 MAPK/NF-κB signaling pathway may promote pro-inflammatory T-cell responses while weakening immune regulatory mechanisms, thereby contributing to sustained immune activation. However, current evidence is mainly derived from EAP models and other inflammatory diseases, and human data on Th17/Treg balance in CP/CPPS are limited and inconsistent (113). Experimental autoimmune prostatitis (EAP), chronic nonbacterial prostatitis (CNP), and human CP/CPPS are related but non-equivalent evidence sources (85).

Whether these models fully recapitulate the complex immune landscape observed in clinical CP/CPPS patients remains controversial. Therefore, future studies integrating single-cell RNA sequencing and spatial transcriptomics will be necessary to further define the cell-type-specific functions of the p38 MAPK/MSK1/NF-κB axis within distinct immune cell populations in human CP/CPPS.

### 4.5 Neuroinflammation and tissue remodeling: involvement of the p38 MAPK/MSK1/NF-κB axis in chronic pain development

A prominent clinical feature of CP/CPPS is persistent pelvic pain, which is not solely caused by local inflammatory injury but also involves complex interactions between the immune and nervous systems (114). Mast cells have emerged as important mediators in this process (115). In men with CP/CPPS, increased mast cell tryptase and nerve growth factor (NGF) have been detected in expressed prostatic secretions (116). In experimental autoimmune prostatitis (EAP) models, increased total and activated mast cells were observed in the prostate, and mast cell-deficient mice showed attenuated pelvic pain behavior without differences in inflammatory infiltrates (116). These findings suggest that mast cells may contribute to pelvic pain through mechanisms partially independent of local inflammatory severity.

Beyond peripheral mechanisms, central neuroimmune activation may also contribute to pain persistence. Sustained activation of inflammatory signaling pathways can promote the production of inflammatory mediators, including TNF-α and IL-1β, and induce the expression of NGF and other neuroinflammatory factors, resulting in sensory nerve sensitization (116). In spinal cord microglia and dorsal root ganglion (DRG) neurons, p38 MAPK is activated in models of persistent pain, and p38 inhibition reduces mechanical allodynia (117). In the superficial spinal dorsal horn, MSK1 contributes to inflammatory pain via epigenetic and transcriptional alterations, including histone H3 phosphorylation (53). However, these findings derive from non-prostatic pain models, and direct evidence for MSK1 involvement in CP/CPPS-associated pelvic pain is currently lacking.

In addition, chronic inflammatory stimulation may promote fibroblast activation and extracellular matrix deposition, leading to structural remodeling of prostate tissue (118). The p38 MAPK/NF-κB signaling pathway has been shown to participate in TGF-β-associated fibrotic responses in various fibrotic diseases (119). In prostate tissue, p38-MAPK signaling has been implicated in stromal remodeling and suppression of androgen receptor expression (120). However, whether this pathway contributes to CP/CPPS-associated tissue fibrosis through MSK1-mediated regulatory mechanisms remains to be elucidated.

Therefore, the p38 MAPK/MSK1/NF-κB axis may contribute to the transition of CP/CPPS from a localized inflammatory condition to a chronic pain syndrome by integrating inflammatory responses, immune dysregulation, and neurotissue alterations, but this remains a hypothesis. The proposed mechanisms and supporting evidence regarding the involvement of this axis in CP/CPPS are summarized in **Table 2**.

**Table 2:** p38 MAPK/MSK1/NF-κB axis-mediated inflammatory amplification network in CP/CPPS.

Biological process	Key molecules/pathways	Proposed mechanism	Evidence source	Current evidence status
Innate immune activation	TLR4/MyD88, IRAK, TRAF6, TAK1, MKK3/6, MKK4, p38 MAPK	Recognition of PAMPs/DAMPs activates TLR4/MyD88 signaling, leading to TAK1-dependent activation of p38 MAPK via MKK3/6 and MKK4	Prostatitis models; non-prostatic inflammatory systems	Supported by inflammatory models; specific triggers in CP/CPPS require further study
Cytokine amplification	TNF-α, IL-1β, IL-6	NF-κB activation promotes inflammatory cytokine transcription; cytokines act as autocrine/paracrine signals to reinforce p38 MAPK activation	CP/CPPS animal models; human seminal cytokine data	Supported by CP/CPPS animal models and heterogeneous human data; causal role not established
Oxidative stress amplification	NOX, ROS	NF-κB-driven inflammatory responses promote ROS production; ROS further activates p38 MAPK/NF-κB signaling	Inflammatory models; oxidative stress studies in CP/CPPS	Supported by inflammatory models and CP/CPPS oxidative stress studies; direct feedback loop not validated
NLRP3 inflammasome cooperation	NLRP3, pro-IL-1β, caspase-1, IL-1β, IL-18	NF-κB primes NLRP3 and pro-IL-1β; ROS and mtDNA promote inflammasome assembly; IL-1β further activates MAPK/NF-κB	Non-prostatic inflammatory systems; NLRP3 detected in expressed prostatic secretions	NLRP3 components detected in CP/CPPS; functional activation and causal contribution require validation
mtDNA-DAMP feedback	cGAS-STING, TLR9, NLRP3, p38 MAPK, NF-κB	ROS-induced mitochondrial damage releases mtDNA, which activates cGAS-STING, TLR9, and NLRP3, further enhancing p38/NF-κB activation	Non-prostatic inflammatory systems	Not directly validated in CP/CPPS; priority for future investigation
Immune microenvironment remodeling	Macrophages (M1/M2), Th17/Treg	p38 MAPK/NF-κB signaling influences macrophage polarization and T-cell balance	EAP models; other inflammatory diseases	Evidence mainly derived from EAP models; human CP/CPPS data limited and inconsistent
Neuroinflammation and pain	Mast cells, NGF, spinal microglia, DRG, MSK1, H3S10ph	Mast cells and NGF contribute to pelvic pain; p38 is activated in spinal microglia and DRG; MSK1 contributes to inflammatory pain in superficial spinal dorsal horn	EAP models; non-prostatic pain models	Mainly EAP model evidence; direct MSK1-pain link in CP/CPPS not established
Tissue remodeling	TGF-β, p38 MAPK/NF-κB, fibroblasts	Chronic inflammation promotes fibroblast activation and extracellular matrix deposition	Prostate tissue studies; fibrotic disease models	p38-MAPK implicated in stromal remodeling; MSK1-mediated fibrosis not established
MSK1-mediated inflammatory memory (hypothetical)	MSK1, H3S10ph, H3S28ph, chromatin accessibility	Persistent activation may maintain inflammatory gene responsiveness through chromatin regulation	Non-prostatic inflammatory systems	Hypothetical; requires re-challenge designs, ATAC-seq/ChIP-seq, and MSK1/2 manipulation

## 5 Therapeutic strategies and translational perspectives targeting the p38 MAPK/MSK1/NF-κB inflammatory amplification network

The therapeutic challenges of chronic prostatitis/chronic pelvic pain syndrome (CP/CPPS) are not solely attributable to the presence of inflammation, but rather to the establishment of a self-sustaining network involving inflammatory signaling, oxidative stress, and immune dysregulation (121). Therefore, simply reducing individual inflammatory mediators or alleviating local symptoms may be insufficient to fundamentally reverse disease progression. Based on the proposed p38 MAPK/MSK1/NF-κB inflammatory amplification model, future therapeutic strategies may require integrated interventions targeting three major aspects: inhibition of key regulatory nodes, disruption of inflammatory feedback loops, and remodeling of the immune microenvironment. Importantly, none of the strategies discussed below has established clinical efficacy in CP/CPPS, and current management remains multimodal.

### 5.1 Direct targeting of core p38 MAPK/MSK1/NF-κB signaling nodes

The p38 MAPK/MSK1/NF-κB axis represents a candidate regulatory hub involved in inflammatory signal transmission and transcriptional control. In CP/CPPS, p38 MAPK activation has been observed in prostatic tissue in some rodent models, and its inhibition by natural compounds such as calycosin has been associated with reduced inflammatory cytokines (63). However, these findings are preliminary, and MSK1 remains largely uninvestigated in prostate tissue (122).

p38 MAPK inhibitors can suppress downstream inflammatory transcriptional programs by blocking p38 phosphorylation and activation, thereby reducing the production of pro-inflammatory cytokines, including TNF-α, IL-1β, and IL-6 (43). Several p38 inhibitors have been evaluated in preclinical and clinical studies, including SB203580, SB202190, BIRB796, and losmapimod (43). In multiple inflammatory disease models, p38 MAPK inhibition has demonstrated anti-inflammatory and tissue-protective effects (123). In the context of CP/CPPS, preclinical studies have demonstrated that pharmacological inhibition of p38 MAPK (e.g., SB203580) or suppression of p38 signaling by natural compounds reduces inflammatory mediator production in rodent models (43, 63). However, no p38-targeted therapy has been evaluated in clinical trials specifically for CP/CPPS, and p38 inhibitors have shown limited clinical efficacy in other chronic inflammatory diseases.

However, p38 MAPK is not exclusively a pathological signaling molecule but also participates in physiological processes, including cellular stress responses, immune defense, and tissue repair (14). Therefore, prolonged and systemic inhibition of p38 MAPK may impair normal immune functions (124). Moreover, previous studies in inflammatory diseases have shown that although p38 MAPK inhibition can reduce inflammatory biomarkers, clinical efficacy has not consistently met expectations, suggesting the existence of complex compensatory pathways within chronic inflammatory networks (125). The p38–MK2 branch may compensate for p38 inhibition, and MK2 inhibitors such as CC-99677 have been developed to target this downstream node (126, 127).

NF-κB, as a central transcriptional regulator of inflammatory gene expression, represents another potential therapeutic target (128). Inhibition of NF-κB activation can simultaneously reduce the expression of multiple inflammatory mediators and limit immune cell recruitment (129). However, because NF-κB is broadly involved in maintaining host immune homeostasis, non-specific blockade may disrupt normal immune functions (130). Therefore, future NF-κB-targeted therapies should focus on cell-specific regulation and localized delivery strategies (131).

Compared with direct inhibition of p38 MAPK or NF-κB, targeting MSK1 may provide a potentially more precise therapeutic strategy. MSK1 inhibitors such as SB-747651A have been characterized in inflammatory models, including macrophage inflammatory responses and neutrophil recruitment (50). However, MSK1 has dual roles in inflammatory regulation. It promotes inflammatory transcription through p65 Ser276 phosphorylation and chromatin remodeling, but it also limits inflammation via DUSP1/IL-10 feedback (19). Therefore, targeting MSK1 requires careful evaluation of its net effect, and selective inhibition may be beneficial only in contexts where its pro-inflammatory functions dominate over its anti-inflammatory feedback roles (50). Importantly, MSK1 is not a p38-exclusive effector, and MSK2 can partially compensate for MSK1 (19). MSK1/2 double-knockout mice are hypersensitive to endotoxic shock, and systemic MSK1 inhibition could remove protective feedback and potentially worsen inflammation (19). In addition, H89 was originally characterized as a “selective” inhibitor of the nucleosomal response and was widely used as an MSK1 tool compound in early studies. However, subsequent profiling revealed that H89 inhibits PKA, S6K1, and ROCK-II with comparable potency, indicating that its selectivity is limited (132). MSK1-targeted therapy in CP/CPPS remains a theoretical concept, and its therapeutic potential requires further experimental validation.

### 5.2 Combined therapeutic strategies targeting the inflammatory–oxidative stress feedback network

Because inflammatory responses and oxidative stress are mutually reinforcing processes in CP/CPPS, interventions targeting a single inflammatory pathway may be insufficient to completely disrupt disease persistence. Therefore, simultaneous regulation of inflammatory signaling and oxidative stress responses may represent a more effective therapeutic strategy.

On the one hand, activation of the p38 MAPK/MSK1/NF-κB axis promotes inflammatory cytokine production and enhances NOX-associated oxidative activity, resulting in sustained ROS accumulation (94); On the other hand, ROS can further activate p38 MAPK and NF-κB signaling, thereby establishing a positive feedback loop characterized by “inflammatory activation–oxidative damage–secondary inflammatory activation” (133). Therefore, combined suppression of inflammatory signaling and enhancement of antioxidant defense mechanisms may achieve superior network-level regulatory effects compared with isolated anti-inflammatory or antioxidant approaches, but this remains to be tested in CP/CPPS.

Among these strategies, enhancement of nuclear factor erythroid 2-related factor 2 (Nrf2)-mediated antioxidant defense may have considerable therapeutic potential. Nrf2 regulates the expression of multiple antioxidant genes and enhances cellular ROS-scavenging capacity, thereby reducing oxidative stress-induced inflammatory signaling activation (134). Several Nrf2 activators have been evaluated in preclinical and clinical studies, including dimethyl fumarate (approved for multiple sclerosis and psoriasis), bardoxolone methyl (CDDO-Me), and sulforaphane (135). However, bardoxolone methyl was associated with increased heart failure risk in the BEACON trial, leading to early termination, which limits its clinical development (136). Theoretically, simultaneous inhibition of the p38 MAPK/NF-κB axis and activation of the Nrf2 pathway may reduce both inflammatory output and oxidative damage-associated feedback amplification (137). Although Nrf2 activators such as sulforaphane have shown efficacy in experimental autoimmune prostatitis models, no Nrf2-targeted therapy has been evaluated in CP/CPPS clinical trials (138).

NOX inhibitors represent another candidate approach for disrupting the inflammatory–oxidative feedback loop. GKT137831 (setanaxib), a first-in-class NOX1/4 inhibitor, has demonstrated anti-inflammatory and anti-fibrotic effects in preclinical models (139). In a phase 2 randomized controlled trial in patients with primary biliary cholangitis, setanaxib did not meet its primary endpoint but showed preliminary evidence for potential anti-cholestatic and anti-fibrotic effects (140). A separate phase 2 trial evaluated GKT137831 in patients with type 1 diabetes and persistently elevated urinary albumin excretion, with the primary outcome being change in albuminuria (141). In prostate-related inflammation, NOX4-derived ROS has been implicated in fibroblast-to-myofibroblast differentiation and stromal remodeling, providing a rationale for NOX inhibition in prostate disease (142). However, NOX inhibitors have not been tested in CP/CPPS, and their therapeutic potential in this context remains speculative.

Furthermore, considering the role of the NLRP3 inflammasome in inflammatory amplification, combined modulation of NF-κB/NLRP3 signaling may represent another candidate therapeutic direction (143). NF-κB mediates the transcriptional priming of inflammasome-related genes, whereas danger signals such as ROS promote inflammasome activation (99). Several NLRP3 inhibitors have been developed, including MCC950, dapansutrile (OLT1177), and inzomelid (144). MCC950 has shown efficacy in preclinical models of autoinflammatory and neuroinflammatory diseases but was discontinued in a clinical study due to liver enzyme elevations (145). Dapansutrile and inzomelid have completed early-phase clinical trials in other inflammatory conditions (146). However, no NLRP3-specific inhibitor has been tested in CP/CPPS, although natural compounds such as juglone and naringin, which inhibit NLRP3 inflammasome activation, have shown efficacy in experimental autoimmune prostatitis models (143). Systemic NLRP3 inhibition carries potential risks, as NLRP3 plays a dual role in host defense against infection (147).

From a translational perspective, prostate-local delivery may reduce the risk of systemic immunosuppression and improve therapeutic precision. Nanocarrier-based delivery systems, including liposomes, polymeric nanoparticles, and hydrogels, have been explored for local treatment of prostate inflammation in preclinical models (148). However, these approaches remain preclinical, and no delivery platform has been validated for CP/CPPS in clinical settings. Therefore, combined targeting of inflammatory transcriptional activation and inflammasome activation may provide a candidate approach to disrupting the chronic inflammatory cycle in CP/CPPS, but current evidence remains hypothesis-generating and no strategy has established clinical efficacy.

### 5.3 Innovative therapeutic perspectives based on the hypothesis of MSK1-mediated inflammatory memory

Conventional anti-inflammatory therapies primarily focus on reducing inflammatory activity; however, the chronic persistence and recurrence of CP/CPPS suggest that disease progression may involve regulatory mechanisms beyond transient inflammatory activation. The recently proposed concept of “inflammatory memory” suggests that prolonged inflammatory stimulation can induce stable epigenetic and transcriptional alterations, allowing inflammation-associated genes to maintain a heightened responsiveness to subsequent stimuli (68). In addition to enhancing NF-κB transcriptional activity, MSK1 can phosphorylate histone H3 at Ser10 and Ser28, a mechanism that has been linked to increased chromatin accessibility at inflammation-associated gene loci in non-prostatic systems (149). Whether this mechanism operates in prostate tissue remains unknown. Therefore, targeting MSK1 may interfere with the mechanisms responsible for maintaining chronic inflammatory states, although the net effect of MSK1 inhibition on ongoing inflammation may depend on the balance between its pro-inflammatory transcriptional functions and its anti-inflammatory negative feedback roles.

However, it should be emphasized that the involvement of MSK1-mediated inflammatory memory in CP/CPPS remains a theoretical hypothesis. Currently, direct evidence demonstrating that MSK1 contributes to persistent chromatin accessibility or long-term activation of inflammation-associated genes in patient tissues or animal models is still lacking. Future studies integrating epigenomic approaches, including chromatin immunoprecipitation sequencing (ChIP-seq) and assay for transposase-accessible chromatin sequencing (ATAC-seq), will be necessary to determine whether MSK1 participates in the establishment of inflammatory memory in CP/CPPS. If validated, MSK1-targeted intervention may represent a therapeutic strategy distinct from conventional anti-inflammatory approaches, shifting the therapeutic goal from simply reducing inflammatory levels toward modifying the fundamental mechanisms underlying disease persistence. Current therapeutic strategies and their potential advantages and limitations are summarized in **Table 3**.

**Table 3:** Therapeutic strategies targeting inflammatory amplification pathways in CP/CPPS.

Target	Therapeutic strategy	Representative agents	Expected effect	CP/CPPS evidence	Current limitations
p38 MAPK	p38 MAPK inhibitors	SB203580, SB202190, BIRB796, losmapimod	Reduce inflammatory signaling and cytokine production	Preclinical rodent models only; no clinical trials in CP/CPPS	Limited clinical efficacy in other chronic inflammatory diseases; toxicity; compensatory pathways
MK2	MK2 inhibitors	CC-99677	Block p38 downstream cytokine production	No CP/CPPS data	p38–MK2 branch compensation; early clinical stage
NF-κB	NF-κB pathway inhibition	Cell-specific and localized delivery strategies	Suppress inflammatory gene transcription	No CP/CPPS-specific data	Broad inhibition may interfere with physiological immune functions
MSK1	MSK1-targeted intervention	SB-747651A; H89 (non-selective)	Potentially inhibit transcriptional amplification downstream of p38 MAPK	No CP/CPPS-specific validation	MSK2 redundancy; MSK1/2–DUSP1/IL-10 protective feedback; systemic inhibition may worsen inflammation
Oxidative stress	Antioxidant intervention	Nrf2 activators: dimethyl fumarate, CDDO-Me, sulforaphane	Reduce ROS accumulation and restore redox balance	Sulforaphane tested in EAP models; no CP/CPPS clinical trials	Antioxidant therapy alone may not block inflammatory amplification
NOX	NOX inhibitors	GKT137831 (setanaxib)	Reduce NOX-derived ROS	Preclinical prostate-related inflammation data only	No CP/CPPS data; phase 2 trials in PBC and diabetic kidney disease
NLRP3	NLRP3 inhibitors	MCC950, dapansutrile, inzomelid	Block inflammasome activation and IL-1β/IL-18 release	Natural compounds (juglone, naringin) in EAP models	MCC950 discontinued due to liver enzyme elevations; no CP/CPPS-specific inhibitor tested; dual role in host defense
Combination therapy	Multi-target regulation of inflammatory and oxidative pathways	p38/MK2 + Nrf2; NF-κB + NLRP3; nanocarrier-based delivery	Break inflammation–oxidative stress feedback loop	No CP/CPPS clinical data	Optimal combination strategy remains unknown
Local delivery	Nanocarrier-based prostate-local delivery	Liposomes, polymeric nanoparticles, hydrogels	Reduce systemic immunosuppression and improve precision	Preclinical prostate inflammation models only	No delivery platform validated in CP/CPPS clinical settings

All strategies: no established clinical efficacy in CP/CPPS.

### 5.4 From single-target therapy to precision regulation of inflammatory networks: future research directions

Due to the substantial heterogeneity of CP/CPPS, single-target therapeutic approaches may be insufficient to address the diverse pathological mechanisms among different patient populations (150). Future studies should adopt a systems-level perspective to elucidate the functions of the p38 MAPK/MSK1/NF-κB axis across different cell types and disease stages.

First, single-cell RNA sequencing and spatial transcriptomics should be applied to identify the specific cellular populations in which this signaling axis is predominantly activated and to clarify the interactions among different cell subsets within the inflammatory microenvironment. Second, integration of proteomic and epigenomic approaches will be necessary to further determine whether MSK1 contributes to inflammatory persistence and disease recurrence mechanisms. Furthermore, nanodelivery systems and local targeted therapeutic strategies may provide new opportunities to improve treatment precision. For example, localized delivery of inflammatory signaling inhibitors may reduce the risk of systemic immunosuppression while enhancing therapeutic safety and efficacy. In addition, structure-based modification of natural products, such as ginkgolide B derivatives with antiplatelet and anti-inflammatory activities, represents a potential strategy for developing new anti-inflammatory agents, although their relevance to CP/CPPS remains untested (151).

Overall, future therapeutic strategies for CP/CPPS may require a conceptual shift from conventional “inflammation suppression” toward “inflammatory network remodeling.” Modulating the p38 MAPK/MSK1/NF-κB axis and its associated feedback circuits, with careful consideration of the dual pro- and anti-inflammatory roles of MSK1, may represent a promising direction for disrupting the vicious cycle of chronic inflammation. Major knowledge gaps and future research directions are summarized in **Table 4**.

**Table 4:** Knowledge gaps and future perspectives.

Current limitation	Key unanswered question	Suggested approaches	Validation standard
Lack of direct MSK1 evidence in CP/CPPS	Is MSK1 activated in patient tissues?	Clinical samples, phosphoproteomics	Detection of p-MSK1 and p-p65 Ser276 in human prostate tissue
Unknown cellular specificity	Which cells activate the p38/MSK1/NF-κB axis?	Single-cell RNA sequencing, spatial transcriptomics	Cell-type-resolved activation maps in CP/CPPS tissue
Unclear inflammatory memory mechanism	Does MSK1 induce persistent chromatin accessibility?	ATAC-seq, ChIP-seq, re-challenge designs, MSK1/2 manipulation	Persistent accessibility after stimulus withdrawal
Limited biomarker studies	Can pathway activation predict disease severity or treatment response?	Biomarker validation studies	Correlation with NIH-CPSI, UPOINT, and inflammatory phenotypes
Therapeutic uncertainty	Can combined inhibition disrupt chronic inflammatory loops?	Preclinical intervention studies	Efficacy in immune-active CP/CPPS models
mtDNA-TLR9/STING link unvalidated	Does mtDNA drive p38/NF-κB activation in CP/CPPS?	Direct measurement of mtDNA release, TLR9 and cGAS-STING activation	Detection in human CP/CPPS tissue and relevant models
No phenotype-stratified therapy	Which patients are most likely to benefit?	Biomarker-stratified patient selection	Prospective enrichment by inflammatory biomarkers

## 6 Conclusion and future perspectives

The development and progression of chronic prostatitis/chronic pelvic pain syndrome (CP/CPPS) are unlikely to be driven by abnormalities in individual inflammatory mediators alone, but rather result from a complex inflammatory network involving innate immune activation, oxidative stress imbalance, and remodeling of the immune microenvironment. However, the molecular mechanisms responsible for the establishment and long-term maintenance of persistent inflammation in CP/CPPS remain incompletely understood.

In this review, we propose that the p38 MAPK/MSK1/NF-κB signaling axis may represent a candidate regulatory hub connecting innate immune sensing, inflammatory transcriptional regulation, and oxidative stress amplification. Under persistent pathological stimulation, activation of this axis may enhance NF-κB-dependent inflammatory gene expression, promote the production of pro-inflammatory mediators such as TNF-α, IL-1β, and IL-6, and may contribute to oxidative stress generation, although this feedback loop has not been directly validated in CP/CPPS. In addition, MSK1, as an important molecular bridge linking p38 MAPK signaling with chromatin regulation, may contribute to the long-term maintenance of inflammatory transcriptional programs, representing a testable hypothesis for the epigenetic basis of chronic persistence and recurrence in CP/CPPS. However, it should be emphasized that MSK1/2 can also exert anti-inflammatory effects through DUSP1 and IL-10, and MSK1/2 double-knockout mice are hypersensitive to endotoxic shock. Therefore, the net effect of targeting this axis may depend on the balance between its pro-inflammatory transcriptional functions and its anti-inflammatory negative feedback roles, and cell-type-specific and context-dependent strategies will be required.

Although direct evidence demonstrating aberrant activation of the p38 MAPK/MSK1/NF-κB axis in patients with CP/CPPS remains limited, findings from inflammatory disease models and related cellular studies support this axis as a promising candidate network for mechanistic investigation and therapeutic exploration. Future studies integrating single-cell sequencing, spatial transcriptomics, epigenomic profiling, and conditional genetic manipulation models will be essential to define the cell type-specific functions and disease-stage-dependent roles of this signaling network.

From a therapeutic perspective, future interventions for CP/CPPS may require a transition from conventional single-target anti-inflammatory approaches toward precision regulation of inflammatory networks. Integrated modulation of the p38 MAPK/MSK1/NF-κB axis, oxidative stress defense systems, and immune microenvironment remodeling may provide new opportunities to disrupt chronic inflammatory cycles. Among these potential targets, MSK1, as a key regulator linking inflammatory signaling with transcriptional and epigenetic reprogramming, may represent a promising direction for future precision therapies in CP/CPPS. However, given the dual pro- and anti-inflammatory roles of MSK1/2, therapeutic targeting will require careful consideration of cell-type-specific and context-dependent effects to preserve protective negative feedback mechanisms.

In conclusion, the p38 MAPK/MSK1/NF-κB-mediated inflammatory amplification network provides a novel conceptual framework for understanding CP/CPPS chronicity and offers potential opportunities for identifying new biomarkers and therapeutic targets. Nevertheless, this model remains to be validated through further clinical investigations and experimental studies, which will be essential for translating this theoretical framework into practical clinical applications.
